# Vasopressin and terlipressin in adult vasodilatory shock: a systematic review and meta-analysis of nine randomized controlled trials

**DOI:** 10.1186/cc12640

**Published:** 2013-06-19

**Authors:** VGM Pereira, A Serpa Neto, SO Cardoso, JA Manetta, DC Espósito, M de Oliveira Prado Pasqualucci

**Affiliations:** 1 ABC Medical School (FMABC), Príncipe De Gales, Santo André, SP, Brazil

## Introduction

Catecholamines are the most used vasopressors in vasodilatory shock. However, the development of adrenergic hypo-sensitivity and the subsequent loss of catecholamine pressor activity necessitate the search for other options. Our aim was to evaluate the effects of vasopressin and its analogue terlipressin compared with catecholamine infusion alone in vasodilatory shock.

## Methods

Systematic review and meta-analysis of publications between 1966 and 2011. The Medline and CENTRAL databases were searched for studies on vasopressin and terlipressin in critically ill patients. The metaanalysis was limited randomized controlled trials evaluating the use of vasopressin and/or terlipressin compared with catecholamine in adult patients with vasodilatory shock. The assessed outcomes were: overall survival, changes in the hemodynamic and biochemical variables, a decrease of catecholamine requirements, and adverse events.

## Results

Nine trials covering 998 participants were included. A metaanalysis using a fixed-effect model showed a reduction in norepinephrine requirement among patients receiving terlipressin or vasopressin infusion compared with control (standardized mean difference, -1.58 (95% CI, -1.73 to -1.44); *P <*0.0001). Overall, vasopressin and terlipressin, as compared with norepinephrine, reduced mortality (relative risk (RR): 0.87 (0.77 to 0.99); *P *= 0.04). Vasopressin compared with norepinephrine decreased mortality in adult patients (RR: 0.87 (0.76 to 1.00); *P *= 0.05) and in patients with septic shock (42.5% vs. 49.2%, respectively; RR, 0.87 (95% CI, 0.75 to 1.00); *P *= 0.05; number needed to treat, 1 to 15). There was no difference in adverse events between the vasopressin and control groups (RR: 0.98 (0.65 to 1.47); *P *= 0.92).

## Conclusion

Vasopressin use in vasodilatory shock is safe, associated with reduced mortality, and facilitates weaning of catecholamines. In patients with septic shock, vasopressin compared with norepinephrine may also decrease mortality.

